# *In Vivo* Imaging of Local Inflammation: Monitoring LPS-Induced CD80/CD86 Upregulation by PET

**DOI:** 10.1007/s11307-020-01543-3

**Published:** 2020-09-28

**Authors:** Marco F. Taddio, Claudia A. Castro Jaramillo, Peter Runge, Alain Blanc, Claudia Keller, Zeynep Talip, Martin Béhé, Nicholas P. van der Meulen, Cornelia Halin, Roger Schibli, Stefanie D. Krämer

**Affiliations:** 1 Center for Radiopharmaceutical Sciences ETH, PSI and USZ, Institute of Pharmaceutical Sciences, Department of Chemistry and Applied Biosciences, ETH Zurich, Vladimir-Prelog-Weg 4, 8093 Zurich, Switzerland; 2 Pharmaceutical Immunology, Institute of Pharmaceutical Sciences, Department of Chemistry and Applied Biosciences, ETH Zurich, Zurich, Switzerland; 3 Center for Radiopharmaceutical Sciences ETH, PSI and USZ, Paul Scherrer Institute (PSI), Villigen, Switzerland; 4 Laboratory of Radiochemistry, Paul Scherrer Institute (PSI), Villigen, Switzerland

**Keywords:** Co-stimulatory molecules, CD80, CD86, PET, Abatacept, Lipopolysaccharides, Molecular imaging

## Abstract

**Purpose:**

The co-stimulatory molecules CD80 and CD86 are upregulated on activated antigen-presenting cells (APC). We investigated whether local APC activation, induced by subcutaneous (s.c.) inoculation of lipopolysaccharides (LPS), can be imaged by positron emission tomography (PET) with CD80/CD86-targeting ^64^Cu-labelled abatacept.

**Procedures:**

Mice were inoculated s.c. with extracellular-matrix gel containing either LPS or vehicle (PBS). Immune cell populations were analysed by flow cytometry and marker expression by RT-qPCR. ^64^Cu-NODAGA-abatacept distribution was analysed using PET/CT and *ex vivo* biodistribution.

**Results:**

The number of CD80^+^ and CD86^+^ immune cells at the LPS inoculation site significantly increased a few days after inoculation. CD68 and CD86 expression were higher at the LPS than the PBS inoculation site, and CD80 was only increased at the LPS inoculation site. CTLA-4 was highest 10 days after LPS inoculation, when CD80/CD86 decreased again. A few days after inoculation, ^64^Cu-NODAGA-abatacept distribution to the inoculation site was significantly higher for LPS than PBS (4.2-fold). Co-administration of unlabelled abatacept or human immunoglobulin reduced tracer uptake. The latter reduced the number of CD86^+^ immune cells at the LPS inoculation site.

**Conclusions:**

CD80 and CD86 are upregulated in an LPS-induced local inflammation, indicating invasion of activated APCs. ^64^Cu-NODAGA-abatacept PET allowed following APC activation over time.

**Electronic supplementary material:**

The online version of this article (10.1007/s11307-020-01543-3) contains supplementary material, which is available to authorized users.

## Introduction

The co-stimulatory molecules CD80 and CD86 are expressed on activated antigen-presenting cells (APCs) including dendritic cells (DCs), macrophages and neutrophils and can be upregulated on B and T cells [1–8]. While CD80 is virtually absent on non-activated APCs, CD86 is constitutively expressed at low levels. The interaction of CD80 or CD86 with CD28 on T cells, together with the signalling between MHC molecules and the T cell receptor, leads to full activation of CD4^+^ T cells which is important for induction of potent CD8^+^ T cells and antibody responses. After the activation phase, upregulated T cell surface protein cytotoxic T lymphocyte-associated protein 4 (CTLA-4) can compete with CD28 for CD80/CD86 binding and induce their trans-endocytosis, terminating T cell co-stimulation [1–5].

CD80 and CD86 are targeted in therapy to downregulate APC activation by blocking the binding of CD80/CD86 to CD28. Abatacept is used for the treatment of rheumatoid arthritis [9]. It is a fusion protein consisting of the extracellular part of CTLA-4 and the Fc part of human IgG1. It lacks both antibody-dependent cell-mediated and complement-dependent cytotoxicity due to modifications in the IgG1 Fc region [10]. Belatacept differs from abatacept by two amino acids in the CD80/CD86 binding site and is used in transplantation medicine to reduce transplant rejection [11].

CD80 and CD86 serve as markers of APC activation in flow cytometry and immunohistochemistry. A CD80/CD86-selective tracer for positron emission tomography (PET) or single-photon emission computed tomography (SPECT) may allow the non-invasive imaging of APC activation. This could be of interest for staging infections, Toll-like receptor-4 (TLR-4)-induced inflammation, autoimmune processes, transplant rejection and for monitoring the effects of cancer immunotherapy [12–19]. Our group has recently evaluated ^111^In-DOTA-belatacept and small molecule inhibitors of human CD80 (hCD80) as SPECT and PET tracers, respectively, for imaging CD80/CD86 in a CD80-positive xenograft mouse model [20, 21].

In the current study, we evaluate CD80/CD86 imaging by PET as a non-invasive method to follow the course of APC activation in a more physiological model of local inflammation. We used the mouse model described by Temme *et al*., where a local inflammation is induced by subcutaneous (s.c.) deposition of lipopolysaccharides (LPS) suspended in extracellular matrix proteins (Matrigel^®^) [14]. LPS activate(s) APCs by signalling via TLR-4 which significantly increases CD80/CD86 expression in monocytes [22–25]. We used radiolabelled abatacept rather than belatacept in this study, as abatacept has a stronger affinity to murine CD80 than belatacept (at similar affinity to murine CD86) [26]. The results from PET and tracer *ex vivo* biodistribution analyses were compared with flow cytometry and RT-qPCR data for CD80-positive (CD80^+^) and CD86^+^ immune cells, as well as CD80, CD86, CD68 and CTLA-4 expression.

## Material and Methods

### Animal Model

Animal experiments were in accordance with the Swiss Animal Welfare legislation and approved by the Veterinary Office of the Canton Zurich (ZH17/2015 and ZH28/2018). All applicable institutional and/or national guidelines for the care and use of animals were followed. Male C57BL/6 mice were purchased from Charles River Laboratories (Sulzfeld, Germany) and housed under a 12-h light/dark cycle with both standard rodent chow and water *ad libidum*. Mice were housed for at least 7 days for adaptation before experiments. Based on the procedure reported by Temme *et al*. [14], 10 μl PBS alone or PBS containing 5 mg/ml LPS from *Salmonella typhimurium* (Calbiochem, CA, USA; 50 μg LPS per animal) were mixed with 40 μl Matrigel Basement Membrane Matrix (Corning™ 354234 from Corning Inc, Corning, NY, USA) and injected s.c. on the right shoulder. Supporting Table S1 shows group sizes for each condition.

### Flow Cytometry

**Fig. 1.**
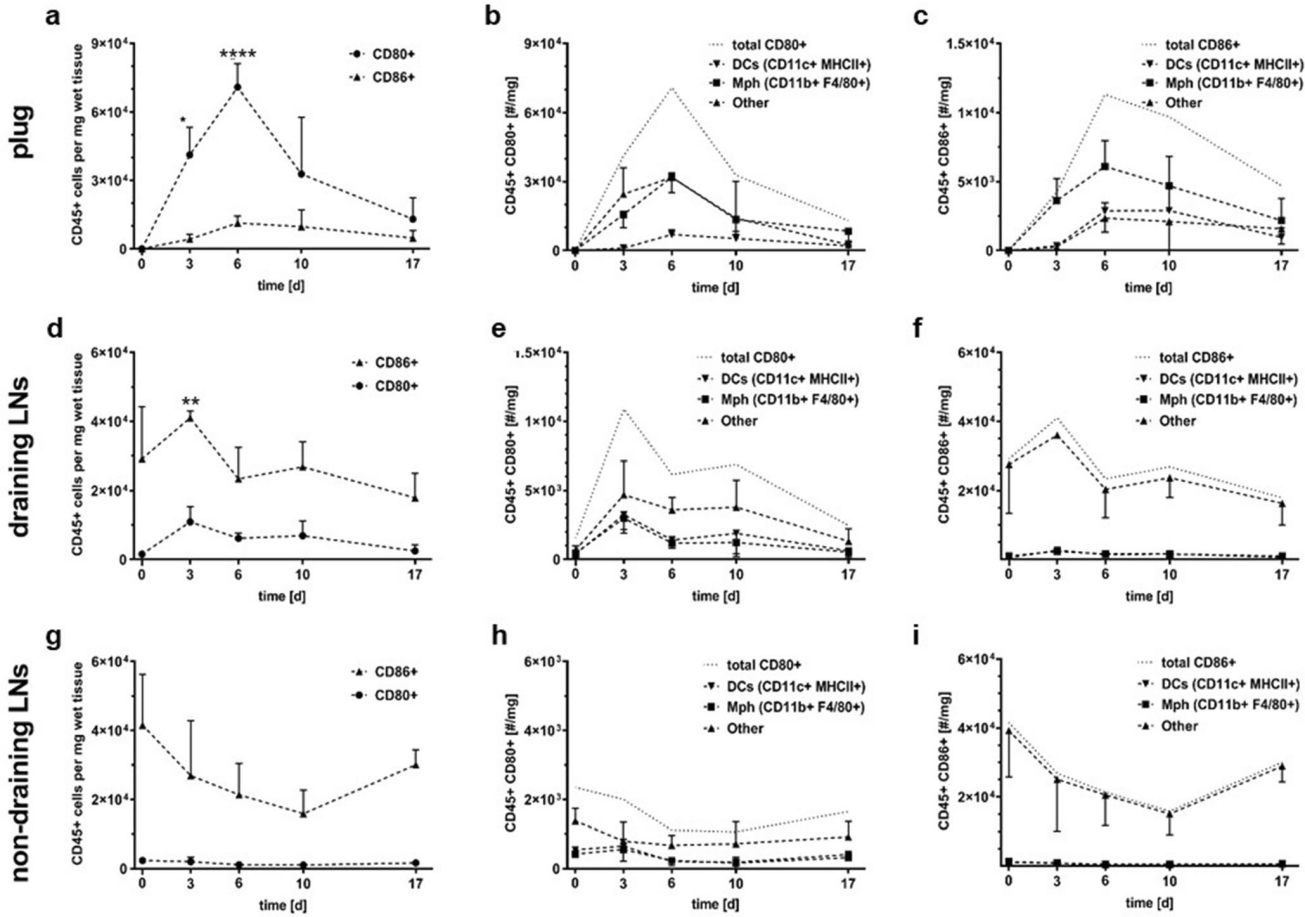
Numbers of CD80^+^ and CD86^+^ leukocytes (CD45^+^) per milligramme wet tissue in LPS/Matrigel plugs and LNs of C57BL/6 mice. Time points after LPS/Matrigel s.c. inoculation as indicated. Day 0, mice without inoculation (cell numbers for plugs were set to 0). Cells were isolated from LPS/Matrigel plugs (**a**–**c**), draining (ipsilateral brachial) LNs (**d**–**f**) and non-draining (contralateral inguinal) LNs (**g**–**i**). **a**, **d**, **g** Numbers of CD80^+^ and CD86^+^ cells. **b**, **e**, **h** Analysis of the CD80^+^ subpopulation. **c**, **f**, **g** Analysis of the CD86^+^ subpopulation. Subpopulations were classified into DCs (CD11c^+^/MHCII^+^), macrophages (CD11b^+^/F4/80^+^) and ‘other’ cell types. Experimental data averaged from 3 mice per time point (except Day 0 with 2 mice), with SD. Number of CD80 and CD86 cells per milligramme wet tissue were compared with the respective numbers 17 days after inoculation by two-way ANOVA with post hoc Dunnet’s test and respective adjustment of *p* values; *p < 0.05; **p < 0.0005; ****p < 0.0001. Note that the CD80^+^ to CD86^+^ cell number ratios were not reproducible between two different experimental settings (compared with Fig. 6).

Dissected plugs were prepared for flow cytometry analysis as described by Temme *et al*. [14]. In brief, single cell suspensions were obtained by shredding the plugs in PBS on ice. Shredded tissue was digested with collagenase D type IV (2 mg/ml; ThermoFisher, MA, USA) and DNAse I from bovine pancreas (100 μl/ml; Sigma-Aldrich, Buchs, Switzerland) at 37 °C for 30 min and passed through a 70 μm cell strainer. Cells were not permeabilised. Lymph nodes (LN) were passed through a 70-μm cell strainer without digestion. The produced cell suspensions were centrifuged at 1200 rpm for 5 min at 4 °C and resuspended in FACS buffer (PBS with 2 % FCS and 2 mM EDTA). The FACS panel consisted of the following antibody-fluorophore conjugates from Biolegend (San Diego, CA, USA): anti-mCD45-PerCP (30-F11), anti-CD80-PE (16-10A1), anti-CD86-APC (PO3), anti-CD11c-PE/Cy7 (N418), anti-CD11b-FITC (M1/70), anti-F4/80-APC/Cy7 (BM8; Fig. 1) or anti-mF4/80-PE/Cy5 (EMR1, Ly71; Fig. 6), anti-mLy6G/Ly6C-Pacific Blue (RB6-8C5), anti-MHCII-BV421 (M5/114.15.2), Zombie-Aqua Fixable Viability Kit. Flow-Count Fluorospheres (Beckman Coulter, Brea, CA, USA) were added to processed tissue samples as internal reference for cell number quantification. All measurements were carried out on a CytoFLEX (Beckman Coulter) using CytExpert Software. Flow cytometry data was analysed with the software FlowJo (www.flowjo.com, BD Life Sciences, Ashland, OR, USA).

### RT-qPCR

Tissues were frozen in liquid nitrogen immediately after dissection, measured in a γ-counter (for biodistribution data; Wizard, PerkinElmer, Waltham, USA) and after decay of the radioactivity used for RT-qPCR analysis. Total RNA was isolated according to the protocols of the Isol-RNA Lysis Reagent (5 PRIME, Gaithersburg, MD, USA). cDNA was generated with the QuantiTect Reverse Transcription Kit (Qiagen, Hilden, Germany). Primers for qPCR were custom-made oligonucleotides from Microsynth (Balgach, Switzerland): murine actin, beta (ACTB; GenBank accession no. NM_007393.3), forward 5′-GTGACGTTGACATCCGTAAAGA-3′, reverse 5′-GCCGGACTCATCGTACTCC-3′; murine CD68 (CD68v2; GenBank accession no. NM_009853), forward 5′-CTGTTCACCTTGACCTGCTCT-3′, reverse 5′-AAACATGGCCCGAAGTGTCC-3′; murine CD80 (CD80; GenBank accession no. NM_009855.2), forward 5′-TGCTGCTGATTCGTCTTTCAC-3′, reverse 5′-GAGGAGAGTTGTAACGGCAAG-3′; murine CD86 (CD86; GenBank accession no. NM_019388), forward 5′-AAAGTTGGTTCTGTACGAGCAC-3′, reverse 5′-GGCCCAGGTACTTGGCATT-3′; CTLA-4 (CTLA-4; GenBank accession no. NM_009843), forward 5′-TTTTGTAGCCCTGCTCACTCT-3′, reverse 5′-CTGAAGGTTGGGTCACCTGTA-3′.

Expression was quantified with the GoTaq® qPCR Kit (Promega, Madison, WI, USA) using a 7900 HT Fast Real-Time PCR System (Applied Biosystems, Carlsbad, CA, USA). The amplification signals were detected in real time, which permitted accurate quantification of the amounts of the initial RNA template during 40 cycles according to the manufacturer’s protocol. All reactions were performed in triplicates, in two independent runs. Quantitative analysis was performed using the 2−ΔΔCt quantification method. The specificity of the PCR products of each run was determined and verified with the SDS dissociation curve analysis feature.

### ELISA

For the quantification of sCD80 in plasma, mice were anaesthetised and euthanised as described under ‘PET/CT Imaging and *Ex Vivo* Biodistribution’. Blood was collected in heparinised tubes and plasma was separated by centrifugation at 4 °C, 1500 × g for 5 min. sCD80 was quantified with the Mouse CD80 DuoSet ELISA (DY740, R&D systems, Minneapolis, MN, USA) according to the manufacturer’s protocol. Fluorescence was measured on a BIO-TEK Synergy HT plate reader (BIO-TEK, Winooski, VT, USA) with excitation at 450 nm and emission detection at 540 nm. Concentrations are expressed as equivalent to the concentration of the standard which has a predicted molecular weight of 51.3 kDa (as communicated by the supplier).

### Surface Plasmon Resonance

Surface Plasmon Resonance (SPR) was performed with amine chips with a dextran surface on a SPR-2 device from Sierra Sensors (Hamburg, Germany). For the immobilisation of protein, a new amine chip was placed in the device under constant flow of PBS with 0.01 % Tween-20 (immobilisation buffer). For preparation of the chip surface, five injection rounds of 100 mM HCl and subsequent injections of immobilisation buffer were run over the coating and the reference spot. To activate the chip surface, 100 μl of a freshly prepared 1:1 mixture of 0.5 M 1-ethyl-3-(3-dimethylaminopropyl)carbodiimid and 0.2 M *N*-hydroxysuccinimid was injected over both spots. A subsequent injection of 20‑40 μl 100 nM rhCD80, rmCD80, rhCD86 from R&D Systems, Minneapolis, MN, USA) over the coating spot resulted in a protein immobilisation level of around 150–250 RU. This was followed by injection of 1 M ethanolamine over both spots to fully deactivate the unreacted chip surface.

The protein-coated chip was kept under steady flow of 50 mM Tris buffer containing 0.01 % Tween-20, pH 7.4 (running buffer). All analytes were diluted with running buffer from a micromolar to a sub-nanomolar range. For the measurement, 100 μl of each dilution were injected with an injection rate of 25 μl/min over 240 s. Data were analysed using Sierra Analyser and Scrubber Software (BiologicSoftware, Canberra, Australia).

### Chelator Conjugation and Radiolabelling

Abatacept (Orencia^®^, Bristol-Myers Squibb, New York, NY, USA; from a local drug store) was conjugated with the chelator SCN-benzyl-1,4,7-triazacyclononane,1-glutaric acid-4,7-acetic acid (*p*-SCN-Benzyl-NODA-GA; Chematech, Dijon, France) according to Cooper *et al*. [27]. Metal-free chemicals for the conjugation were purchased from Sigma-Aldrich (Buchs, Switzerland). The protein to *p*-SCN-Benzyl-NODA-GA ratio for conjugation was 1:20. The produced conjugate (NODAGA-abatacept) was purified by 7 ultracentrifugation steps with Ultra Centrifugal Filter Units (Amicon, 15 ml, 30 kDa cutoff) according to the protocol [27]. The chelator/protein ratio of the product was quantified by deglycosylation with Protein Deglycosylation Mix P6039 (New England Biolabs, Ipswich, MA, USA), reduction with DTT and fragment analysis with LC-MS. Copper-64 was produced via the ^64^Ni(p,n)^64^Cu nuclear reaction at the Injector II cyclotron facility at the Center for Radiopharmaceutical Sciences, Paul Scherrer Institute (CRS, PSI, Villigen, Switzerland) [28].

^64^CuCl_2_ (700 MBq) in 20 μl 0.1 M HCl was mixed with 20 μl 0.5 M ammonium acetate buffer at pH 5.5, before 45 μl NODAGA-abatacept (60 μM, 2.7 nmol) was added at room temperature to produce ^64^Cu-NODAGA-abatacept. The mixture was shaken for 30 min at 37 °C until > 95 % of ^64^Cu(II) was chelated. The chelation process was monitored by radio-HPLC using a MassPrep phenyl guard column (1000 Å, 20 μm, 2.1 mm × 10 mm, 10–500 K, Waters, Baden, Switzerland) with H_2_O/MeCN with 0.1 % TFA as mobile phase with 20 % MeCN to elute the unchelated ^64^Cu(II) and subsequent 75 % MeCN to elute the protein.

### PET/CT Imaging and *Ex Vivo* Biodistribution

^64^Cu-NODAGA-abatacept (25 μg, 5.9–36.4 MBq at time of injection) was injected into a tail vein of the awake, constrained animal. For blocking conditions, additional 1 mg unconjugated abatacept was co-administrated (immediately before the tracer injection). At 48 h post-injection, mice were anaesthetised with 2 % isoflurane/air (1:1). Body temperature was kept at 37 °C with warm air and respiration rate at ~ 60 min^−1^ by adjusting the isoflurane/air flow. PET was acquired for 90 min (static mode) with a subsequent CT scan on a Super Argus PET/CT scanner (Sedecal S.A., Madrid, Spain) with an axial field of view of 4.8 cm and a spatial resolution of 1.6–1.7 mm (full width at half maximum) [29]. PET data were reconstructed by 2D Fourier rebinning/ordered-subset expectation maximisation (FORE/OSEM) with 2 iterations and 16 subsets, correcting for singlets and randoms but not attenuation. The voxel size for image analysis was 0.243 mm in all 3 dimensions. Image data were analysed with the software PMOD v3.9 (PMOD, Zurich, Switzerland) and with the base (version 3.6.1) and oro.nifti (version 0.9.1) packages of the R-project (*R*; www.r-project.org). Immediately after termination of the scans, mice were euthanised by decapitation still under anaesthesia and dissected. Tissue radioactivity was quantified with a *γ*-counter. For both PET and *ex vivo* biodistribution analysis, tissue radioactivity was expressed as standardised uptake value (SUV), which is the decay-corrected radioactivity per cm^3^ or g, divided by the injected radioactivity dose per g body weight, assuming a tissue density of 1 g/cm^3^. Percent injected dose per g tissue (% ID/g) were calculated as SUV/body weight (g) × 100 %, with the average body weight of all mice in the study (22.1 g).

### Statistical Analysis

Shown are mean values with SD. If not stated otherwise, conditions were compared by homoscedastic two-tailed Student’s *t* test (GraphPad Prism version 8.0.0, GraphPad Software, San Diego, CA, USA). Alternatively, two-way ANOVA with Tukey’s test was applied, as indicated (GraphPad Prism). Linear mixed effects modelling was performed with the function lme (package nlme, version 3.1-140) of *R*. Results from lme were compared by the *R* function ANOVA (package stats, version 3.6.1). For all tests, differences at *p* < 0.05 were considered significant.

## Results

### Subcutaneous LPS Induces Infiltration of CD80^+^ and CD86^+^ Cells

Mice were s.c. inoculated with Matrigel containing LPS (LPS/Matrigel) as described in the ‘Material and Methods’ section. On Days 3, 6, 10 and 17 after inoculation, we analysed, using flow cytometry, the numbers of CD80^+^ and CD86^+^ immune cells in the implanted LPS/Matrigel plug and in draining (ipsilateral brachial) and non-draining (contralateral inguinal) LN. For the analysis, single cell suspensions were pre-gated for singlets and living cells, with subsequent gating for leukocytes (CD45^+^ cells). The gating strategy is shown in Supporting Fig. S1. Numbers of CD80^+^ and CD86^+^ immune cells transiently increased in the LPS/Matrigel plugs, peaking on Day 6 (Fig. 1a). A minor fraction of the CD80^+^-gated cells (Fig. 1b) was identified as DCs (CD11c^+^/MHCII^+^), while the majority of the cells were identified as macrophages (CD11b^+^/F4/80^+^) or other cell types. CD86^+^ leukocytes in the LPS/Matrigel plug (Fig. 1c) were mainly macrophages.

Draining and non-draining LNs (Fig. 1d, g) contained constitutively high numbers of CD86^+^ leukocytes. The number of CD80^+^ immune cells transiently increased in the draining but not the non-draining LNs (Fig. 1e, h). The CD80^+^ cell population in the draining LNs included macrophages and DCs along with other cell types, while macrophages and DCs were negligible in the CD86^+^ cell population (Fig. 1f, i). The CD86^+^ cells could be germinal centre B cells [30]. After LPS/Matrigel inoculation, draining LNs had a significantly higher weight and cell count than non-draining LNs (Supporting Fig. S2). Note that cell numbers in Fig. 1 are normalised to tissue weight.

### CD80 as a Specific and Early Marker for LPS-Induced Inflammation

**Fig. 2.**
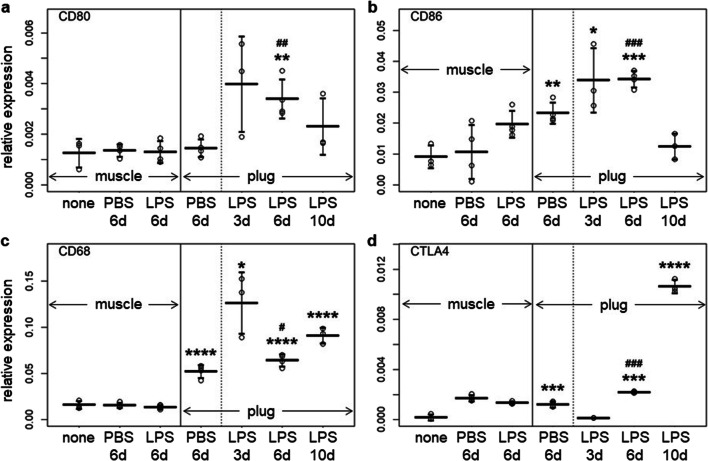
mRNA levels of **a** CD80, **b** CD86, **c** CD68 and **d** CTLA-4 in muscles of untreated C57BL/6 mice (‘muscle none’, *n* = 3 mice) or muscle or plug of LPS/Matrigel (LPS)-inoculated mice on Days 3 (*n* = 3), 6 (*n* = 4) and 10 (*n* = 3) or PBS/Matrigel (PBS)-inoculated mice on Day 6 (*n* = 4) after inoculation, as indicated. Expression levels were quantified by RT-qPCR relative to β-actin. Symbols, individual values; horizontal lines, means; error bars, SD. Significance compared with muscle tissue of untreated mice (indicated with an asterisk) or with PBS/Matrigel plugs, 6 days (indicated with the number symbol) was analysed with student’s *t* tests, without post hoc corrections for multiple comparisons. *^, #^*p* < 0.05; **^, ##^*p* < 0.01; ***^, ###^*p* < 0.005; ****p < 0.001.

To further characterise the inflammation, we analysed mRNA expression levels of CD80, CD86, CD68 and CTLA-4 in LPS/Matrigel plugs 3, 6 and 10 days after inoculation and in PBS/Matrigel plugs (control) 6 days after inoculation. Muscle (ipsilateral quadriceps femoris) of non-inoculated and the inoculated mice were analysed for comparison (Fig. 2). In LPS/Matrigel plugs, expression levels of CD80 and CD86 were increased already at early time points with significant difference to muscle of untreated mice on Days 3 (for CD86) and 6 (for both). Levels were reduced again on Day 10. CD68 was significantly higher in LPS/Matrigel plugs than in muscle of untreated mice from Days 3 to Day 10, indicating migration of cells from myeloid origin (monocytes, macrophages, DCs) into the plug. CTLA-4 was unchanged on Day 3, but increased significantly towards Day 10. All markers had significantly higher levels in the LPS/Matrigel than PBS/Matrigel plugs (6 days after inoculation). The difference was marginal though for CD68. Expression levels in PBS/Matrigel plugs were higher than in muscle of untreated mice, except for CD80 which was virtually absent in PBS/Matrigel plugs. Muscle tissues of the inoculated mice did not reach the marker levels of LPS/Matrigel plugs.

### Production and *In Vitro* Characterisation of ^64^Cu-NODAGA-Abatacept

PET requires a positron-emitting tracer with strong affinity to the imaging target. From the two available CD80/CD86-binding fusion proteins, abatacept has a stronger affinity to recombinant murine CD80 (rmCD80) than belatacept. The two proteins have a similar affinity to rmCD86 which is about one magnitude weaker than the abatacept/rmCD80 interaction [26]. We confirmed by SPR the stronger affinity to rmCD80 of abatacept (*K*_d_ ~ 2 nM) than belatacept (*K*_d_ ~ 12 nM) and chose abatacept for labelling with the radionuclide copper-64. The *K*_d_ of abatacept and belatacept to recombinant human CD80 (rhCD80) and rhCD86 were 0.7 and 0.3 nM, respectively. Binding parameters and exemplary SPR sensograms are shown in Supporting Table S2 and Supporting Fig. S3, respectively.

For the copper-64 labelling process, abatacept was conjugated with the activated chelator NODAGA according to Cooper *et al*. [27] (see ‘Material and Methods’). The protein-to-chelator ratio of the conjugate NODAGA-abatacept was between 0.9 and 1.3. SPR with NODAGA-abatacept confirmed preservation of the affinity to rhCD80 (Supporting Fig. S3b; Supporting Table S2). The labelled conjugate, ^64^Cu-NODAGA-abatacept, had molar activities between 100 and 300 GBq/μmol. ^64^Cu-NODAGA-abatacept was stable at 37 °C in PBS (tested for 48 h) and human plasma (tested for 72 h), respectively (Supporting Fig. S4).

To further evaluate ^64^Cu-NODAGA-abatacept, we performed *in vitro* autoradiography on available tissue slices from human xenografts. Supporting Fig. S5 shows higher binding of ^64^Cu-NODAGA-abatacept to slices of Raji (hCD80^+^ Burkitt’s lymphoma cells) than NCI-H69 (hCD80^-^ small cell lung carcinoma cells) xenografts. Co-incubation with an excess of the small molecule hCD80 inhibitor MT107 [21] or non-conjugated abatacept reduced ^64^Cu-NODAGA-abatacept binding to the Raji slices, indicating that the accumulation of tracer in the hCD80-rich tissue was specific.

### LPS-Induced Inflammation Can Be Imaged with ^64^Cu-NODAGA-Abatacept

**Fig. 3.**
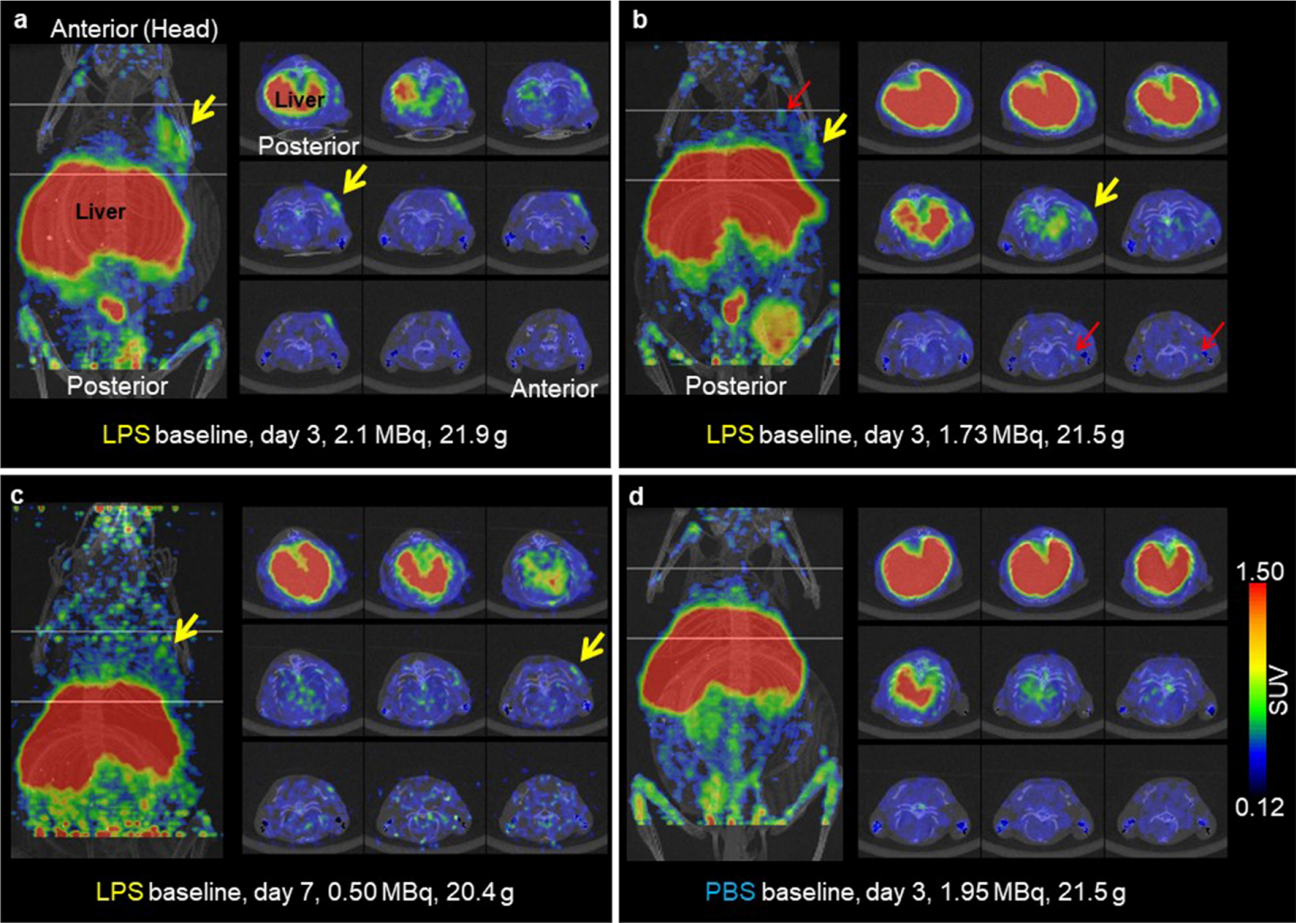
PET/CT images of C57BL/6 mice 48 h after 25 μg ^64^Cu-NODAGA-abatacept i.v. injection. Dose in MBq at scan start and body weight as indicated. Mice were s.c. inoculated with LPS/Matrigel (**a**–**c**) or PBS/Matrigel (**d**) on the right shoulder, 3 days (**a**, **b**, **d**) or 7 days (**c**) before the scan. Maximal intensity projection (MIP), coronal view, on the left of each panel. The region between the two horizontal lines in the coronal MIP (45 voxels apart) are shown as 9 axial segments on the right in each panel. The 9 segments are MIPs of 5 adjacent axial slices each. The segments are shown from posterior (top left) to anterior (bottom right). Yellow arrows, tracer accumulation in the region of inoculation. Red arrows, tracer accumulation in the region of lymph nodes. SUV colour scale, PET standardised uptake value; grey, CT (the respiration sensor is visible besides the skeleton of the mouse). Only scans with visible tracer accumulation in the LPS/Matrigel plugs are shown in (**a**)–(**c**). Accumulation was not visible in several scans after LPS/Matrigel inoculation (see Supporting Figs. S6, S7, S8, S9, S10, S11, S12, S13 and S14). Tracer accumulation was not seen in any of the scans with PBS/Matrigel-inoculated mice (*n* = 6 for 3 days after inoculation; see Supporting Figs. S9 and S13). The SUV ratio plug/opposite side (averaged from 100 voxels with maximal radioactivity each) was 3.2 for the scan in (**a**) and 2.5 for the scan in (**c**).

We conducted PET/CT scans with ^64^Cu-NODAGA-abatacept 3, 7, 10, 17 or 24 days after s.c. inoculation of LPS/Matrigel. Mice inoculated with PBS/Matrigel were scanned 3 or 17 days after inoculation for comparison. For all scans, ^64^Cu-NODAGA-abatacept (25 μg) was injected intravenously (i.v.) 48 h before the scan. PET images showed ^64^Cu-NODAGA-abatacept accumulation in some but not all of the LPS/Matrigel plugs on Days 3 and 7 after inoculation. No accumulation was detected in PBS/Matrigel plugs. PET/CT images of mice with tracer accumulation in the region of LPS/Matrigel inoculation and of a representative PBS/Matrigel-inoculated mouse (3 days after inoculation) are shown in Fig. 3. Supporting Figs. S6, S7, S8, S9, S10, S11, S12, S13, S14 and S16 show images of all PET/CT scans of this study. Quantification from the PET data was challenged by the inter-individual heterogeneity of tracer uptake in the regions of LPS/Matrigel inoculation and by the high scattering due to low radioactivity in some of the scans (see Supporting Figs. S6, S7, S8, S9, S10, S11, S12, S13 and S14). In addition, the plug location was not always obvious. We, therefore, proceeded with the *ex vivo* biodistribution data for quantification.

**Fig. 4.**
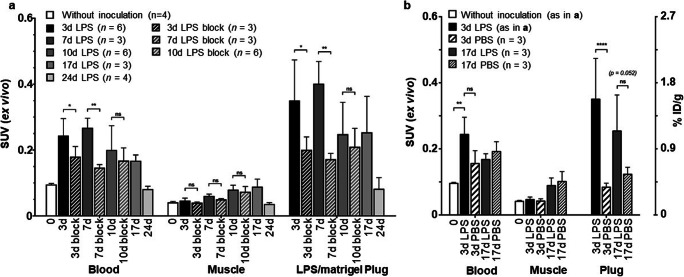
**a** *Ex vivo* biodistribution data of ^64^Cu-NODAGA-abatacept 48 h after i.v. injection of ^64^Cu-NODAGA-abatacept in C57BL/6 mice under baseline (tracer only) and blocking (block, tracer co-administered with 1 mg unconjugated abatacept) conditions. **b** Comparison with mice 3 and 17 days after s.c. PBS/Matrigel inoculation. Means with SD, *n* as indicated. For every inoculation time point and tissue, radioactivity between baseline and blocking or between LPS and PBS were compared by Student’s *t* test, with no post hoc correction for multiple comparisons. For the *t* tests, time points were combined for evaluating the representative variance for the individual tissues. *p < 0.05; **p < 0.005; ****p < 0.0001; ns, not significant.

Figure 4 shows the *ex vivo* biodistribution analysis after dissection. In LPS/Matrigel-inoculated mice, tracer SUV in LPS/Matrigel plug and blood was highest on Days 3 and 7 after inoculation. In muscle tissue, SUV steadily increased from Days 3 to 17. On Day 24, levels in blood and muscle were back to those in mice without inoculation and levels in the LPS/Matrigel plug were similar to the respective blood levels. In addition to the significant uptake in the LPS/Matrigel plugs, accumulation was high in the immune-relevant tissues LNs and spleen, as well as liver (Supporting Fig. S15). Note that SUV = 0.1 corresponds to ~ 0.1 μg tracer/g tissue or ml blood (~ 1 nM).

The SUV from *ex vivo* biodistribution analysis was significantly higher (4.2-fold) in LPS/Matrigel than PBS/Matrigel plugs on Day 3 after inoculation (Fig. 4b). The plug-to-muscle ratio was 7.4 ± 1.6 for LPS/Matrigel-inoculated mice, significantly higher than for PBS/Matrigel-inoculated mice (2.0 ± 0.3). On Day 17, SUV in LPS/Matrigel was 2.1-fold (not significant) higher than in PBS/Matrigel plugs (Fig. 4b). The respective plug-to-muscle ratios were back to 3.0 ± 1.3 and 1.3 ± 0.2.

**Fig. 5.**
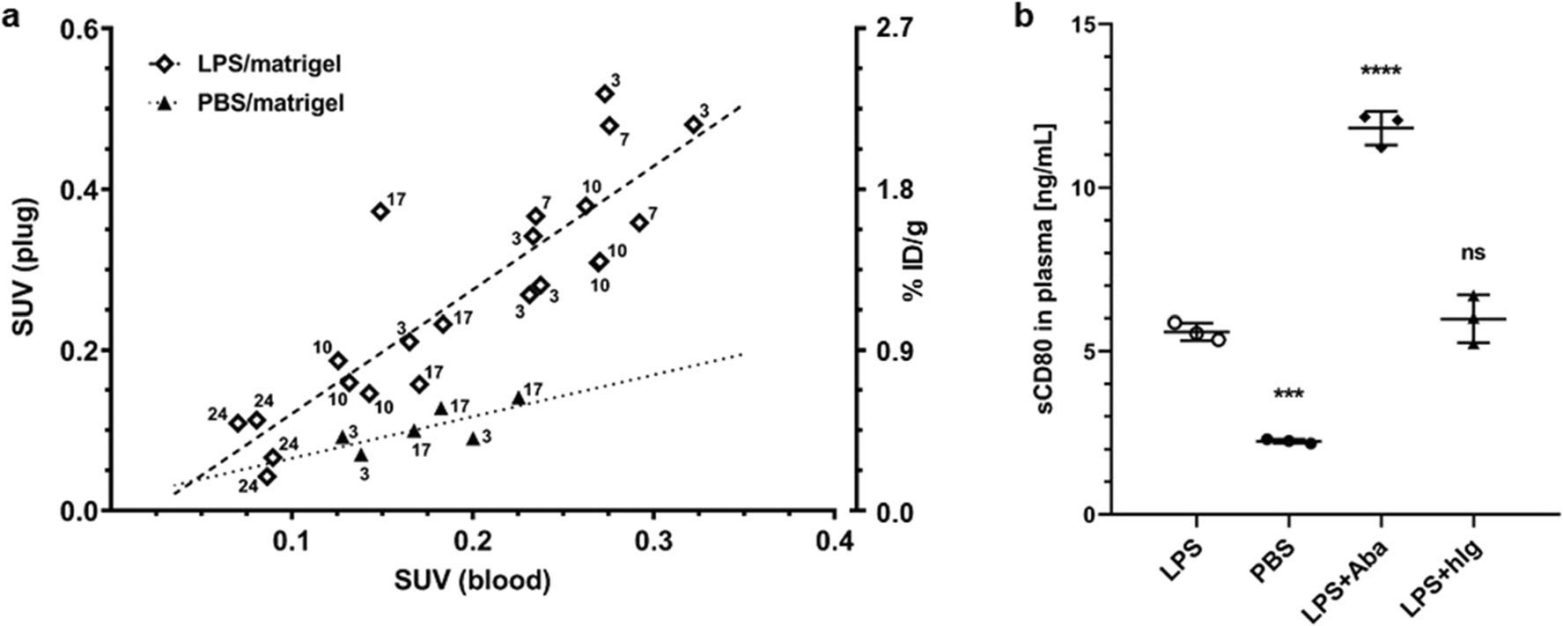
**a** Correlation between plug and blood SUV of ^64^Cu-NODAGA-abatacept from *ex vivo* biodistribution data. Data includes all animals (C57BL/6 mice) with s.c. LPS/Matrigel (open diamonds) and PBS/Matrigel (closed triangles) under baseline conditions (see Fig. 4). Labels indicate days after inoculation. Broken and dotted lines show the linear regressions. Mixed effects modelling revealed a significantly stronger effect of LPS than PBS on the plug-to-blood SUV ratio, independent of whether the intercept was forced to 0 or not (*p* < 0.005). **b** sCD80 levels in plasma samples 3 days after LPS/Matrigel (LPS) or PBS/Matrigel (PBS) inoculation. Inoculated mice were either not further treated or injected with 1 mg abatacept (+Aba) or 1 mg hIg (+hIg) 48 h before blood sampling. Symbols, individual values; horizontal bars, means with SD (*n* = 3 for each condition). Concentrations were calculated for the predicted molecular weight of the assay standard, *i*.*e*., rmCD80 (51.3 kDa). Data were analysed by two-way ANOVA with post hoc Tukey’s test for the factor treatment (LPS, PBS, LPS+Aba, LPS+hIg) and respective adjustment of *p* values; ***p < 0.0005; ****p < 0.0001; ns, not significant, all compared with LPS.

As both plug and blood SUV were higher in LPS than PBS/Matrigel-inoculated mice, we further compared the plug-to-blood ratios to evaluate whether LPS had a significant effect on the distribution of the tracer from blood to the plug. Figure 5 shows the *ex vivo* biodistribution derived SUV of all LPS- and PBS/Matrigel plugs compared with the corresponding SUV of blood. Linear mixed effects modelling relating plug SUV to blood SUV revealed a significantly stronger effect of LPS than PBS on the plug-to-blood SUV ratio. This indicates that the higher SUV in LPS/Matrigel than PBS/Matrigel plugs is not (solely) the result of the higher SUV in blood of LPS/Matrigel-inoculated mice. We cannot exclude, however, that vascularisation and thus tracer availability differed between LPS/Matrigel and PBS/Matrigel plugs.

### LPS Increases sCD80 in Blood

To investigate why ^64^Cu-NODAGA-abatacept in blood was increased after LPS/Matrigel inoculation, we analysed the tracer distribution in blood 7 days after inoculation. ^64^Cu-NODAGA-abatacept was injected i.v. 48 h before the analysis. Assuming a haematocrit of 0.47 [31], the plasma contained (92.3 ± 2.3) % of the blood radioactivity (*n* = 3). This excludes the possibility that the > 2-fold higher blood radioactivity in LPS compared with PBS/Matrigel-inoculated mice resulted from tracer binding to blood cells. We further investigated whether soluble CD80 (sCD80) in plasma could be responsible for the increased blood SUV after LPS/Matrigel inoculation. sCD80 was significantly higher in LPS/Matrigel than PBS/Matrigel-inoculated mice (Fig. 5b). However, the determined sCD80 plasma concentration in LPS/Matrigel-inoculated mice (5.59 ± 0.22 ng/ml) would account for < 10 % of the blood SUV, corresponding to a maximal SUV contribution of ~ 0.01 (assuming 5.59 ng/ml would bind ~ 10 ng/ml ^64^Cu-NODAGA-abatacept, *i*.*e*., aequimolar). We did not determine sCD86 levels in plasma. Taking the weaker binding affinity into consideration, > 50 ng/ml sCD86 would be required to contribute a SUV of 0.1.

### Reduced ^64^Cu-NODAGA-Abatacept Accumulation Under Blocking Conditions

To further investigate whether tracer uptake in the LPS/Matrigel plugs was specific, we compared the accumulation of ^64^Cu-NODAGA-abatacept under baseline conditions and after co-injection of 1 mg unconjugated abatacept to block the binding sites (blocking conditions). Blocking was tested 3, 7 and 10 days after LPS/Matrigel inoculation. The respective PET/CT scans are shown in Supporting Figs. S8, S10 and S12. No tracer accumulation was observed in the plugs under standard experimental conditions with blocking (accumulation was seen under blocking conditions in pilot scans with higher LPS dose, Supporting Fig. 8). Figure 4 and Supporting Fig. S15 show the *ex vivo* biodistribution analyses under baseline and blocking conditions over time. Compared with baseline conditions, co-administration of 1 mg unconjugated abatacept together with ^64^Cu-NODAGA-abatacept resulted in significant reductions of tracer in the LPS/Matrigel plugs and in blood on Days 3 and 7, when baseline SUV were highest. Muscle SUV were reduced by trend under blocking conditions, without significance and to lesser extents than the corresponding LPS/Matrigel plugs.

We tested whether the observed reductions after co-administration of excess abatacept were tissue-dependent, as expected if related to CD80 tissue levels. For the linear mixed effects modelling, SUV ratios between baseline and blocking (Days 3 and 7) were transformed to differences in logarithmic SUV. The interaction between tissue (blood, muscle, LPS/Matrigel plug) and blocking was significant (*p* = 0.0002), indicating that SUV reduction after abatacept co-administration was tissue-dependent and did not just reflect the reduction in blood. With muscle as reference tissue, both the interaction blocking:blood and blocking:plug were significant (*p* < 0.01), with highest impact of plug. With blood as reference, the interaction blocking:plug was close to significance (*p* = 0.058).

Analysing blood distribution of the tracer 7 days after LPS/Matrigel inoculation (48 h after tracer and abatacept i.v. injection), plasma contained (90.3 ± 2.1%) of the radioactivity of whole blood (*n =* 3) with no significant difference to baseline conditions (92.3 %, see section ‘LPS Increases sCD80 in Blood’). This excludes blocking of blood-cell binding as a reason for the reduced radioactivity in blood under blocking conditions. Alternatively, the reduced SUV in blood could result from displacement of ^64^Cu-NODAGA-abatacept from sCD80 in the plasma. However, as discussed in section ‘LPS Increases sCD80 in Blood’, sCD80 levels were too low for a substantial contribution to blood ^64^Cu-NODAGA-abatacept concentration.

### Co-administration of Human Immunoglobulin Reduces CD86^+^ Cells and ^64^Cu-NODAGA-Abatacept Accumulation

**Fig. 6.**
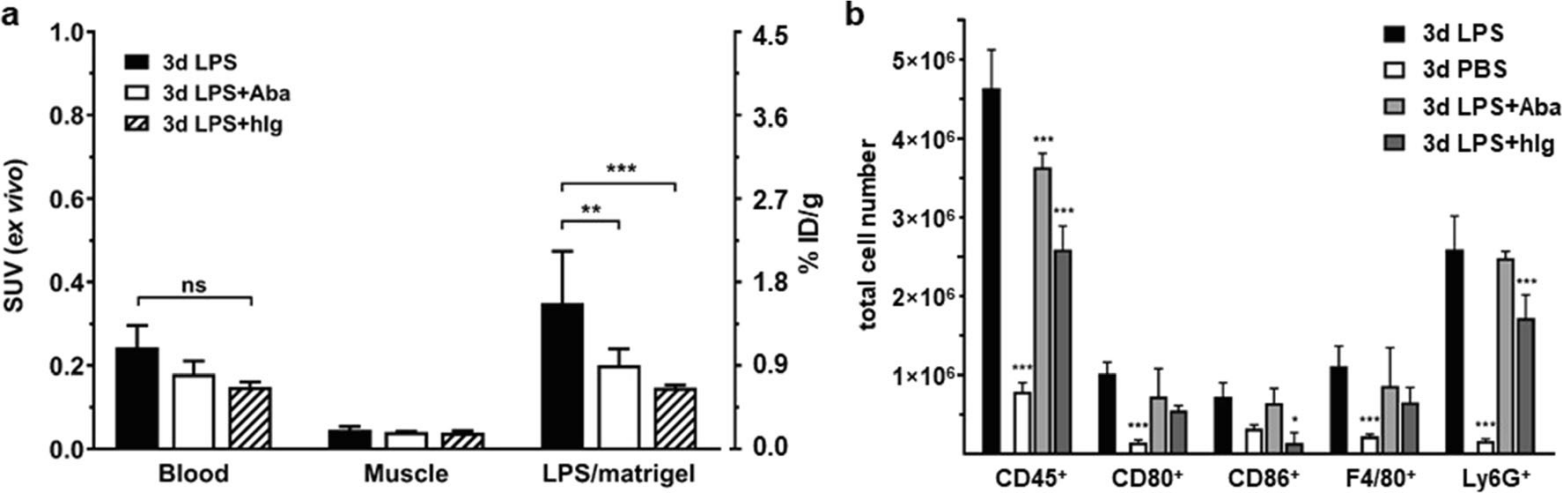
Influence of hIg on ^64^Cu-NODAGA-abatacept distribution and immune cell numbers in LPS/Matrigel plugs of C57BL/6 mice, 3 days after inoculation. **a***Ex vivo* biodistribution 48 h after ^64^Cu-NODAGA-abatacept i.v. injection. Means with SD for baseline (LPS, *n =* 6, same data as in Fig. 4), tracer co-administration of 1 mg unconjugated abatacept (LPS+Aba, *n =* 3, same data as in Fig. 4) and tracer co-administration of 1 mg hIg (LPS+hIg, *n* = 3). **b** Flow cytometry data of Matrigel plugs with total cell numbers of CD45^+^ cells (immune cells) and CD45^+^-gated CD80^+^, CD86^+^, F4/80^+^ (macrophages) and Ly6G/Ly6C^+^ (monocytes, granulocytes, neutrophils) cells under the same conditions as in (**a**) (but different mice) and with PBS/Matrigel (PBS) in addition. Means with SD from 3 mice per condition. Data were analysed by two-way ANOVA with post hoc Tukey’s test for the factor treatment (LPS, PBS, LPS+Aba, LPS+hIg) and respective adjustment of *p* values; *p < 0.05; **p < 0.005; ***p < 0.0005; ns, not significant.

Next, we aimed to assess in an *ex vivo* biodistribution experiment a potential involvement of Fc receptor binding on ^64^Cu-NODAGA-abatacept distribution in LPS/Matrigel-inoculated mice. We intended to block the Fc receptors by co-injection of 1 mg human immunoglobulin (hIg; Gammanorm^®^, Octapharm, Lachen, Switzerland) together with the tracer. This resulted in a similar significant reduction in tracer accumulation in LPS/Matrigel plugs (3 days after inoculation) as co-administration of 1 mg non-conjugated abatacept for blocking (Fig. 6; Supporting Fig. S15). The respective PET/CT scans are shown in Supporting Fig. S16, where no tracer accumulation was visible in the LPS/Matrigel plugs (*n* = 3).

The observed extent of SUV reduction by hIg was unexpected. We hypothesised that the reduced SUV could result from the previously reported immunosuppressive effect of i.v.-administered immunoglobulin [32, 33]. We investigated the influence of hIg and excess abatacept on the numbers of CD80^+^ and CD86^+^ immune cells in LPS/Matrigel plugs, on the frequency distributions of the respective surface densities in the whole cell population, and on plasma sCD80 levels, 3 days after inoculation (48 h after hIg or abatacept i.v. injection). PBS/Matrigel-inoculated mice without treatment were used for comparison. The numbers of total immune cells (CD45^+^) and all tested sub-populations (CD80^+^, CD86^+^, F4/80^+^ as a marker for macrophages, Ly6G/Ly6C^+^ as a marker for monocytes, granulocytes and neutrophils) were higher in the plugs of LPS/Matrigel-inoculated mice than PBS/Matrigel-inoculated mice (not significant for CD86^+^ cells; Fig. 6b; gating strategy in Supporting Fig. 1b). Both abatacept and hIg significantly reduced the number of total immune cells in the LPS/Matrigel plugs. In the case of hIg, reduction was significant and strong for CD86^+^ cells (81 % reduction) and significant but weaker (34 % reduction) for Ly6G/Ly6C^+^ cells (Fig. 6b). A relatively high fraction of CD80^+^ cells in the LPS/Matrigel plugs was Ly6G positive (Supporting Fig. 1b). However, the reduction in CD80^+^ cells by hIg was not significant. Abatacept had no significant effect on the cell numbers of the individual cell sub-populations in the LPS/Matrigel plugs (minor reductions by trend). Regarding the average fluorescence intensity of the CD45^+^CD80^+^ and CD45^+^CD86^+^ cells in Fig. 6b, hIg significantly (*p* < 0.05) reduced the mean (by factor 2.0), median (by factor 2.5) and geometrical mean (by factor 2.1) fluorescence of the CD45^+^CD86^+^ but not CD45^+^CD80^+^ cells in the LPS/Matrigel plugs. In contrast with hIg, abatacept (1 mg) had no effect on the average fluorescence intensity of the CD45^+^CD86^+^ or CD45^+^CD80^+^ cells. The ratios of the means, medians or geometrical means between LPS and LPS+abatacept conditions were all between 0.85 and 1.1. hIg had no effect on sCD80 in the plasma of LPS/Matrigel-inoculated mice while abatacept doubled the sCD80 concentration (Fig. 5b).

## Discussion

Targeting CD80/CD86 with the ^64^Cu-labelled fusion protein abatacept, allowed to follow the activation of APCs in LPS-induced local inflammation using PET and *ex vivo* biodistribution analysis. The time course of increased ^64^Cu-NODAGA-abatacept accumulation at the inflammation site was in agreement with the appearance of CD80^+^ and CD86^+^ immune cells and with CD80 and CD86 expression levels. At later time points, 2–3 weeks after LPS inoculation, tracer uptake decreased again in agreement with the decrease in CD80^+^ and CD86^+^ immune cell numbers and the increased number of CTLA-4^+^ cells at the inflammation site. The fact that numbers of CD80^+^ and CD86^+^ immune cells and CD80 and CD86 expression were higher after LPS/Matrigel than PBS/Matrigel inoculation is in agreement with the TLR-4 related effects of LPS [22–25]. We consider our imaging results, together with the results from the *in vitro* autoradiography with human xenograft slices and the strong affinity of abatacept to hCD80 and hCD86, promising towards the development of CD80/CD86-targeting tracers for clinical imaging.

The use of the fusion protein abatacept, which reaches distribution equilibrium in mice ~ 24 h after an i.v. bolus and has a terminal plasma half-life of ~ 24 h in mice [34, 35], bears several challenges for imaging. In general, distribution should be equilibrated for constant signal ratios between target-rich and reference (low or no target) region. The earliest time point for quantitative imaging after injection of labelled abatacept in mice is thus 24 h. We chose 48 h to guarantee equilibrated distribution and as 48 h provided better imaging results than 18 h in our former study with the related protein ^111^In-DOTA-belatacept [20, 21]. For labelling, we chose Cu-64 as it provides high spatial resolution due to its low decay energy (0.7 mm mean range in water before annihilation) and as ^64^Cu-NODAGA complexes have an excellent stability *in vivo* [36–38]. The physical (radioactivity decay) half-life of 12.7 h resulted in signal loss of > 90 % within the 48 h between tracer injection and imaging. A radio-metal with a longer physical half-life, such as zirconium-89 (physical half-life 78.4 h; 1.3 mm mean range in water before annihilation) would provide a higher residual signal (66 % remaining after 48 h). However, the signal would spill to a ~ 6-fold larger volume (cubic of difference in range in water) which could be of relevance regarding the small size of the plugs.

As a further challenge, biological effects induced by abatacept cannot be excluded within the 48 h between tracer injection and imaging, in particular, under blocking conditions at a dose of 1 mg (~ 40 mg/kg) abatacept. Lorenzetti *et al.* recently found that abatacept reduced surface CD80 (and non-significantly CD86) on stimulated B cells *in vitro* after 2 days in culture [39]. The authors detected internalisation of fluorescently labelled abatacept by B cells after its binding to surface CD80/CD86. Mayer *et al*. observed an abatacept-induced reduction of surface CD80 and CD86 *in vitro* on bone-marrow-derived DCs after LPS treatment. Abatacept was partially internalised by the cells [40]. Within the 48 h between tracer/abatacept administration and imaging in our study, CD80/CD86 surface proteins may thus be reduced and tracer may be internalised. Both challenge the interpretation of the results, in particular comparing baseline with blocking conditions. In our study, 1 mg abatacept did not significantly reduce the numbers of CD80^+^ and CD86^+^ immune cells (reductions by trend) and had no significant effect on their average cell surface density in the LPS/Matrigel plugs 2 days after administration. Based on these findings, we conclude that the reduced SUV after excess abatacept administration resulted from blocking, indicating the specificity of tracer binding *in vivo*.

To shorten the time between tracer administration and imaging and to potentially reduce biological effects which may result in adverse clinical effects besides biasing the imaging results, we are currently evaluating optimised protein tracers (Castro *et al*., unpublished) and are developing small molecule PET tracers selectively targeting hCD80 [21, 41]. ^64^Cu-NODAGA-abatacept binds to both CD80 and CD86 with strong affinity. A CD80-selective tracer would be of advantage. In agreement with state of the art knowledge [2], CD80 was a better indicator of APC activation than CD86 as the latter was constitutively high in LNs and was increased in PBS/Matrigel control plugs, in contrast with CD80.

In contrast with abatacept, hIg administration drastically reduced the number of CD86^+^ immune cells in the LPS/Matrigel plugs and in addition reduced the average cell surface CD86. A reduction in the numbers of CD86^+^ (and CD80^+^) cells after hIg treatment was observed before [32, 33]. The reduced ^64^Cu-NODAGA-abatacept uptake in the LPS/Matrigel plug after hIg administration is in agreement with the reduced number of CD86^+^ immune cells in the plug. We originally intended to use hIg to test for unspecific tracer uptake by Fc receptors. Based on our findings described here, we cannot exclude a contribution of this alternative mechanism to ^64^Cu-NODAGA-abatacept accumulation. We did not find information on whether the mutations which reduce binding to human Fc receptors have the same effect on the binding to murine Fc receptors [10]. The unmodified human IgG1 Fc part would have a weaker affinity to the murine than human Fc receptors [28]. The above mentioned optimised CD80-targeting smaller proteins lack the Fc part. SPECT experiments with ^99m^Tc-labelled smaller protein revealed high accumulation in CD80-positive syngrafts with significant blocking by excess unlabelled protein, confirming Fc-receptor independent uptake (Castro *et al*., unpublished).

We did not find a plausible explanation for the reduced tracer concentration in blood after excess abatacept or hIg administration. We excluded blocking of tracer binding to blood cells or sCD80 as possible reasons. We did not find any indication of dose-dependent general pharmacokinetics, in agreement with published data [34, 35]. The fact that blood SUVs of LPS/Matrigel-inoculated mice under conditions of excess abatacept or hIg were reduced towards SUV of blood from mice with a PBS/Matrigel (control) plug indicates that the increased blood SUV in LPS/Matrigel-inoculated mice was a specific and reversible (by abatacept or hIg) effect of LPS. Potential bias from differences in blood radioactivity may be reduced by comparing tissue ratios rather than absolute values.

Regarding the increased levels of sCD80 3 days after 1 mg abatacept administration, we hypothesise that abatacept binding to sCD80 in plasma reduced its clearance, prolonging its plasma half-life. As discussed, sCD80 levels were too low to be able to affect ^64^Cu-NODAGA-abatacept blood levels.

In conclusion, non-invasive imaging with a CD80/CD86-targeting PET tracer allowed following of APC activation in a model of local inflammation. As an outlook, clinical imaging with an optimised tracer with selectivity for CD80 and shorter plasma half-life may in the future provide information on the state of APC activation in disease and under immunotherapy in oncology.

## Electronic supplementary material

ESM 1 (PDF 6802 kb)

## Abbreviations

APC: antigen-presenting cell
CT: computed tomography
DC: dentridic cell
i.v: intravenous
LN: lymph node
LPS: lipopolysaccharides
NODAGA: 1,4,7-triazacyclononane,1-glutaric acid-4,7-acetic acid
PBS: phosphate-buffered saline
PET: positron-emission tomography
s.c: subcutaneous
SUV: standardised uptake value
