# Supplementary material for: In Vivo Imaging of Local Inflammation: Monitoring LPS-Induced CD80/CD86 Upregulation by PET
Source: Mol Imaging Biol. 2020 Sep 28;23(2):196–207. doi: 10.1007/s11307-020-01543-3 (PMC7910267; doi:10.1007/s11307-020-01543-3)
Supplement: Supplementary file 1 — (PDF 6802 kb) [file 11307_2020_1543_MOESM1_ESM.pdf]

In vivo Imaging of Local Inflammation: Monitoring LPS-Induced CD80/CD86 Upregulation by PET

Supporting Information

Marco F. Taddio[1], Claudia A. Castro Jaramillo[1], Peter Runge[2], Alain Blanc[3], Claudia Keller[1], Zeynep Talip[3], Martin Béhé[3], Nicholas P. van der Meulen[3,4], Cornelia Halin[2], Roger Schibli[1,3], Stefanie D. Krämer[1]

[1] *Center for Radiopharmaceutical Sciences ETH, PSI and USZ, Institute of Pharmaceutical Sciences, Department of Chemistry and Applied Biosciences, ETH Zurich, Switzerland*

[2] *Pharmaceutical Immunology, Institute of Pharmaceutical Sciences, Department of Chemistry and Applied Biosciences, ETH Zurich, Zurich, Switzerland*

[3] *Center for Radiopharmaceutical Sciences ETH, PSI and USZ, Paul Scherrer Institute, Villigen-PSI, Switzerland*

[4] *Laboratory of Radiochemistry, Paul Scherrer Institute, Villigen-PSI, Switzerland*

Correspondence to

marco.taddio@alumni.ethz.ch

stefanie.kraemer@pharma.ethz.ch

Supporting Figures

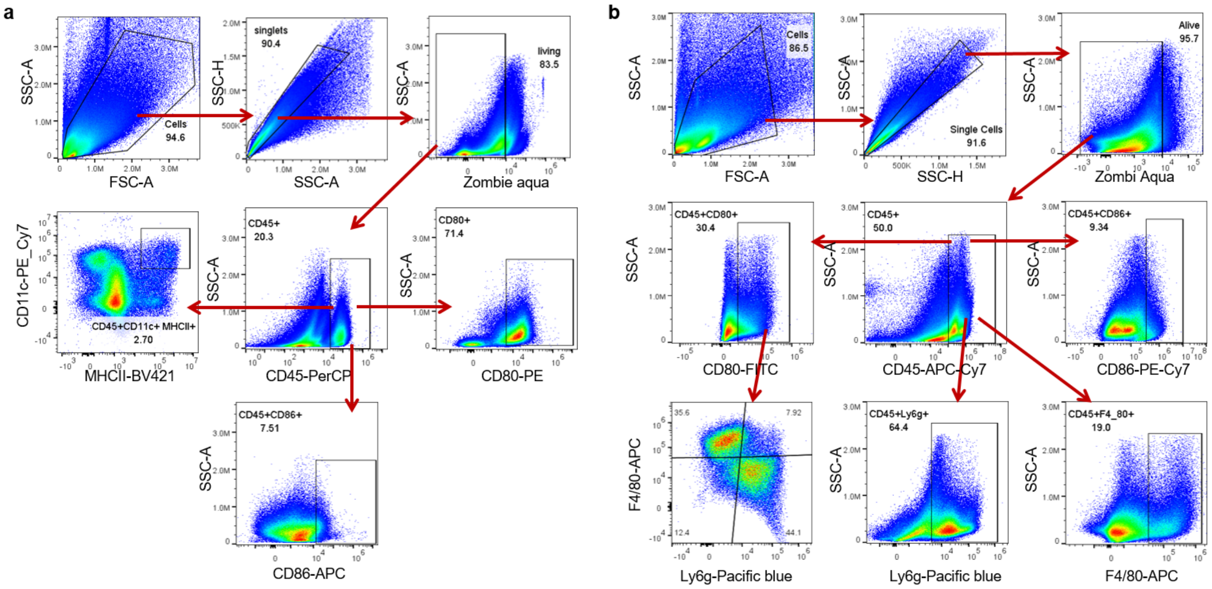

Supporting Figure S1. Full gating strategy for flow cytometry. **a**) For data shown in Figure 1. **b**) For data shown in Figure 6.

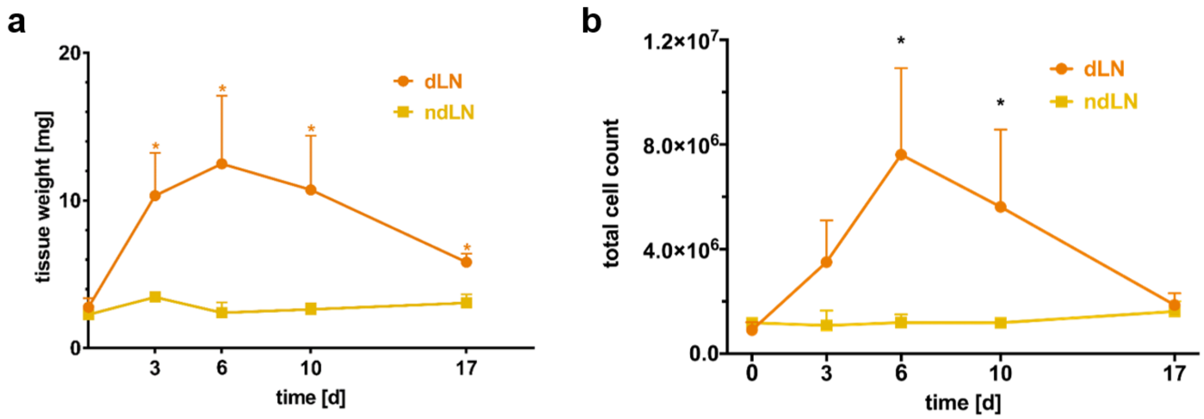

Supporting Figure S2. Weight of wet tissue (**a**) and cell counts (**b**) of draining (proximal brachial) and non-draining (distal inguinal) LNs dissected from LPS/Matrigel-inoculated mice 3, 6, 10 and 17 days after inoculation (n = 3). Day 0, not inoculated. *, $p < 0.05$, comparing draining with non-draining LNs per time point (no correction for multiple comparisons).

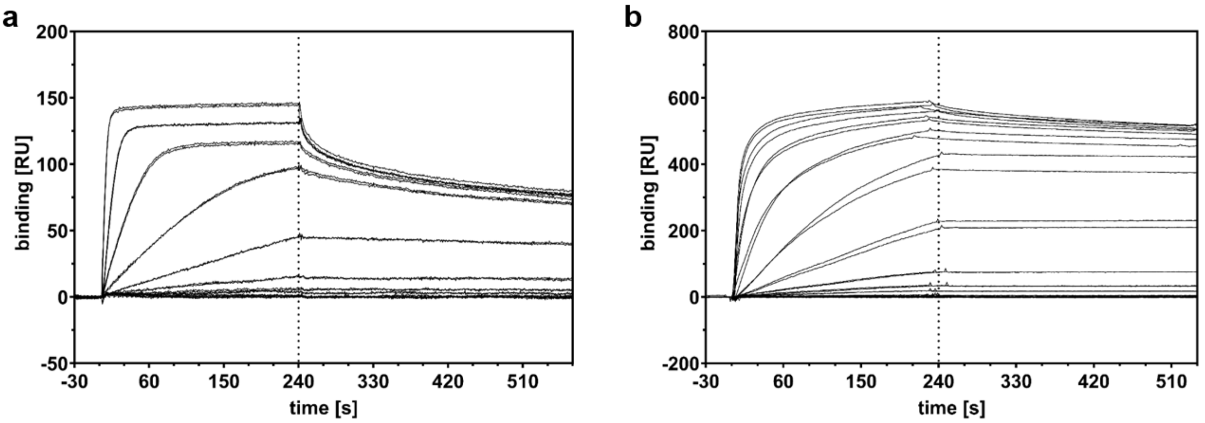

Supporting Figure S3. Representative SPR sensorgrams of **a**) abatacept (3.4 pM – 200 nM) and **b**) NODAGA-abatacept (10 pM – 1.5 µM) binding to immobilized rhCD80. Results are shown in Supporting Table S1. Note that spikes around 0s (± 3s) and at 240s (± 3s) were smoothened.

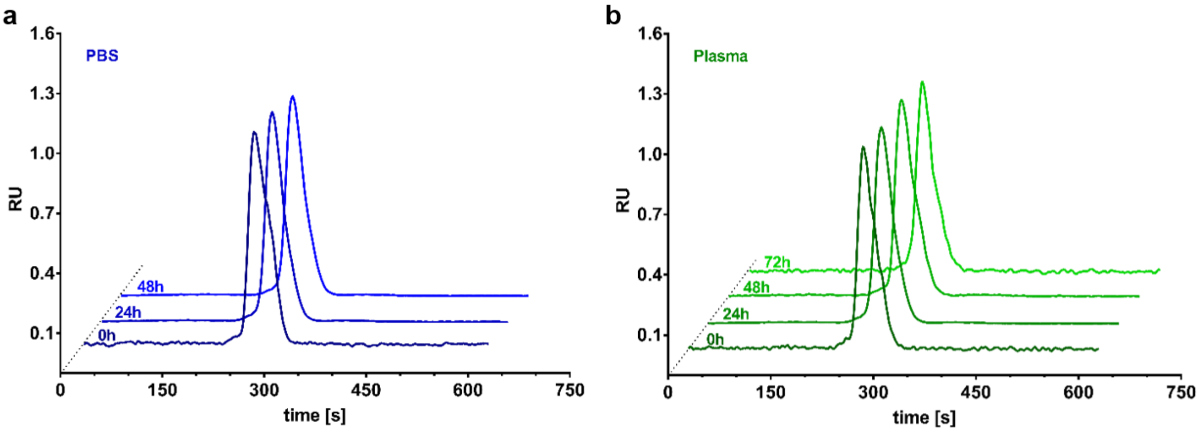

Supporting Figure S4. Chromatograms of ^{64}Cu-NODAGA-abatacept at 37°C after the indicated incubation times in PBS (**a**) and human plasma (**b**). Determined by radio-HPLC coupled to a size exclusion column (Phenomenex, BioSep-SEC-S4000, 300 × 7.8 mm). Chromatograms were normalized to the respective peak maximum. Only parent tracer was detectable.

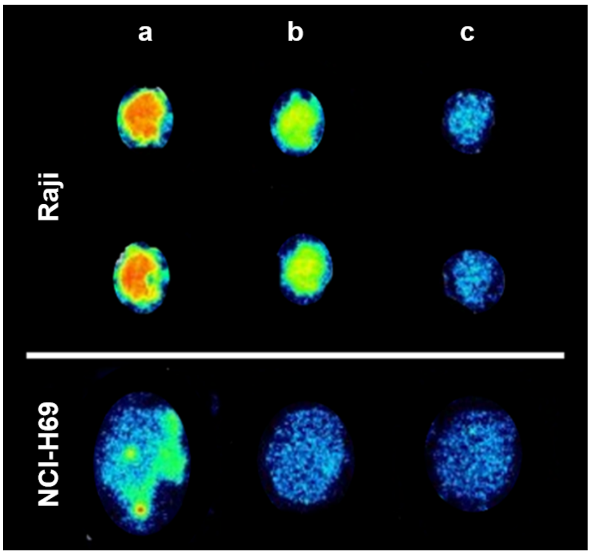

Supporting Figure S5. Autoradiography with slices of human Raji (hCD80/hCD86 positive) and NCI-H69 (NCI; hCD80/hCD86 negative) xenografts incubated with 1 nM ^{64}Cu-NODAGA-abatacept. **a**) Baseline; **b**) incubation together with 10 µM MT107 [18]; **c**) incubation together with 10 µM unconjugated abatacept. Slices were exposed on same plate. Results were confirmed in a second autoradiography experiment.

Method: Tissue slices (20 µm) of hCD80-positive Raji xenografts and hCD80-negative NCI-H69 xenografts, as previously reported in Müller *et al.* [39], were thawed and pre-incubated for 15 min at room temperature with autoradiography buffer (50 mM HEPES, 5 mM $MgCl_2$, 1 mM $CaCl_2$, 125 mM NaCl; pH 7.4) containing 0.1% bovine serum albumin (BSA). The xenograft tissues were incubated with 1 nM ^{64}Cu-NODAGA-abatacept in autoradiography buffer containing 0.1% BSA and 0.33% DMSO for 30 min. For the blocking of specific binding, the incubation solution contained 10 µM MT107 [18] or unconjugated abatacept in addition. To remove unbound tracer, tissues were washed once for 1 min with autoradiography buffer containing 0.1% BSA, twice for 1 min each with autoradiography buffer without BSA and twice for 10 seconds with distilled water. The slices were subsequently dried under a constant air flow, exposed to an imaging screen for 3 h and read out on a BAS-5000 analyser (Fujifilm, Tokio, Japan).

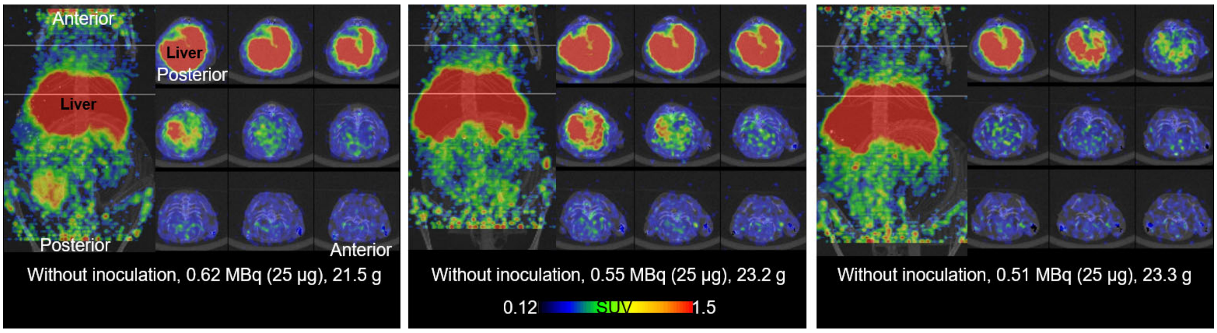

Supporting Figure S6. PET/CT images of C57BL/6 mice 48 h after 25 µg ^{64}Cu-NODAGA-abatacept i.v. injection. Mice without inoculation. Dose in MBq at scan start and body weight as indicated. Note that heads are not included in the field of view. For further details, see Figure 3 in the manuscript.

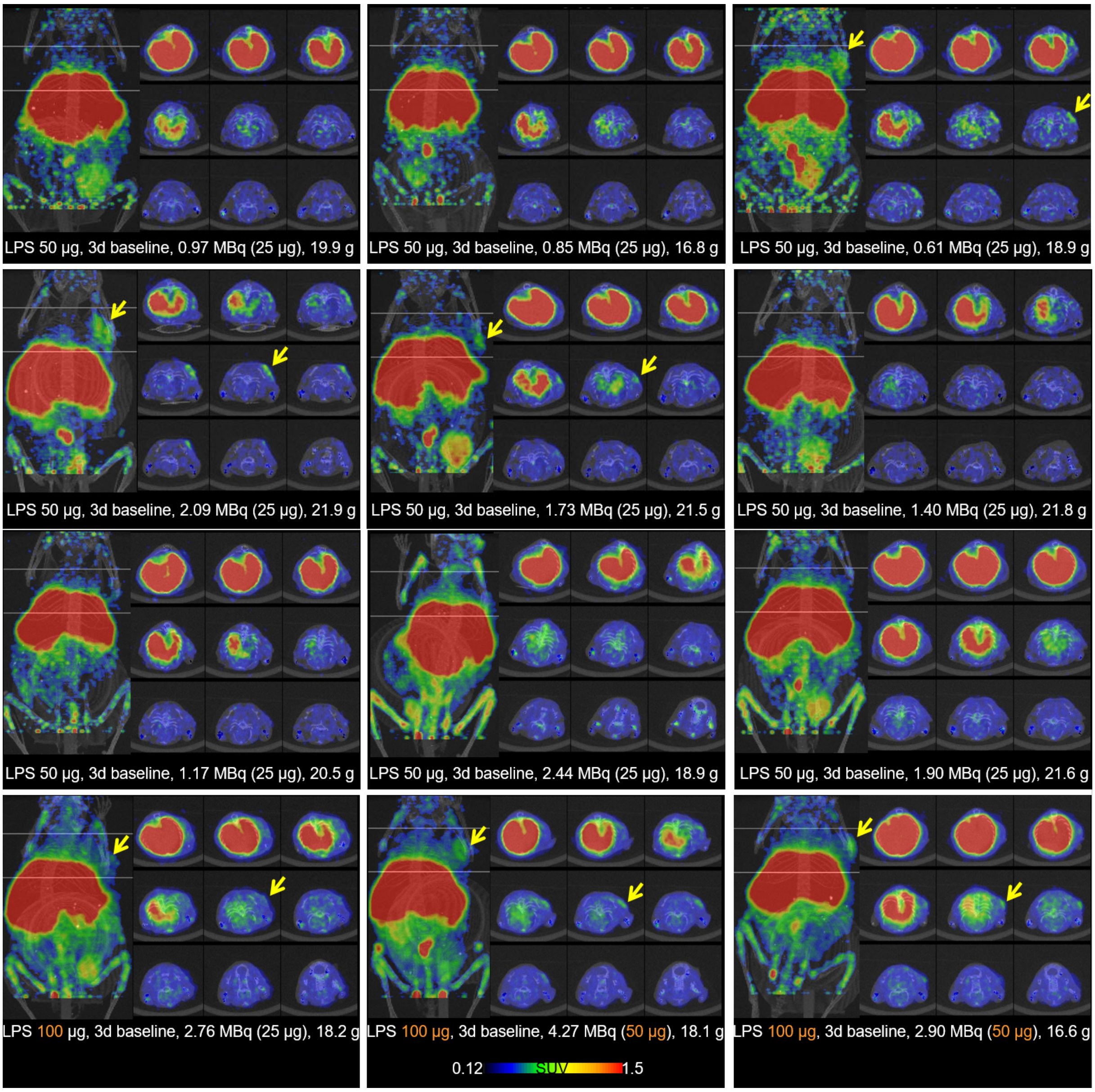

Supporting Figure S7. ^{64}Cu-NODAGA-abatacept PET/CT images 3 d after s.c. LPS/Matrigel (50 or 100 µg LPS as indicated) inoculation on the right shoulder. ^{64}Cu-NODAGA-abatacept was i.v. injected 48 h before scan start. Dose in MBq at scan start (with corresponding mass in µg) and body weight as indicated. Yellow arrows, tracer accumulation in the region of LPS/Matrigel inoculation. For further details, see Figure 3 in the manuscript. The pilot scans in the first and last row were not included in the main study (manuscript) as the LPS source differed from the main study (first row) or LPS and/or tracer dose differed (indicated in orange text).

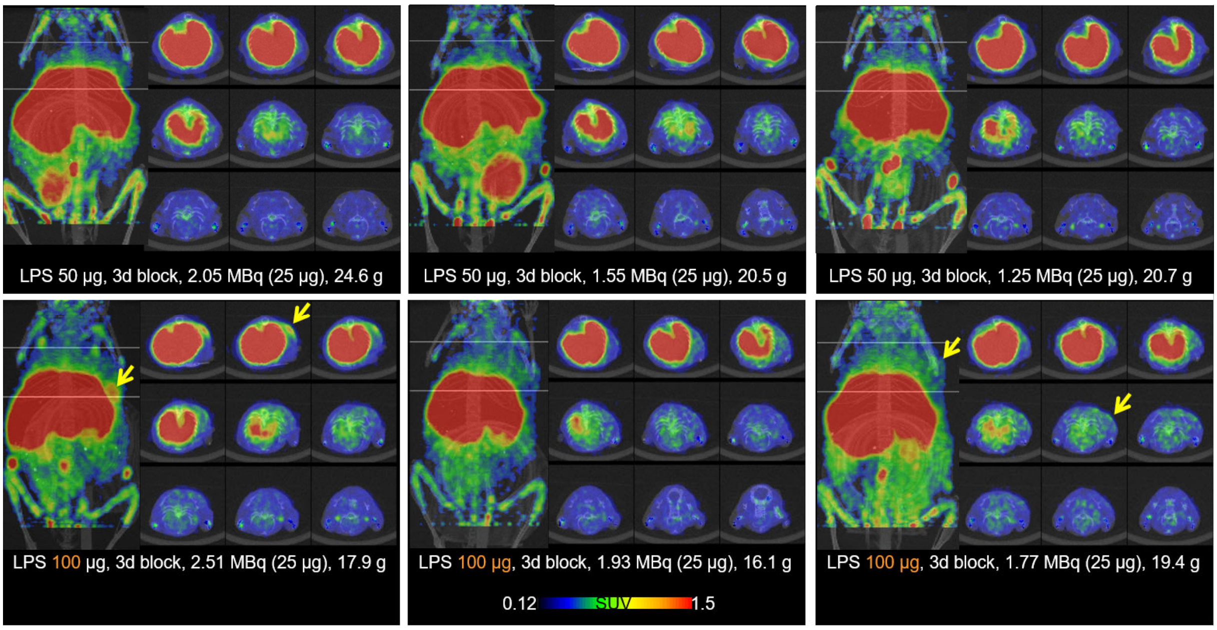

Supporting Figure S8. ^{64}Cu-NODAGA-abatacept PET/CT images 3 d after s.c. LPS/Matrigel inoculation on the right shoulder, under blocking conditions. ^{64}Cu-NODAGA-abatacept was i.v. injected 48 h before scan start together with 1 mg unconjugated abatacept for blocking ("block"). Dose in MBq at scan start and body weight as indicated. Yellow arrows, tracer accumulation in the region of LPS/Matrigel inoculation. For further details, see Figure 3 in the manuscript. The pilot scans in the bottom row were not included in the main study (manuscript) as the LPS dose was higher (indicated in orange text).

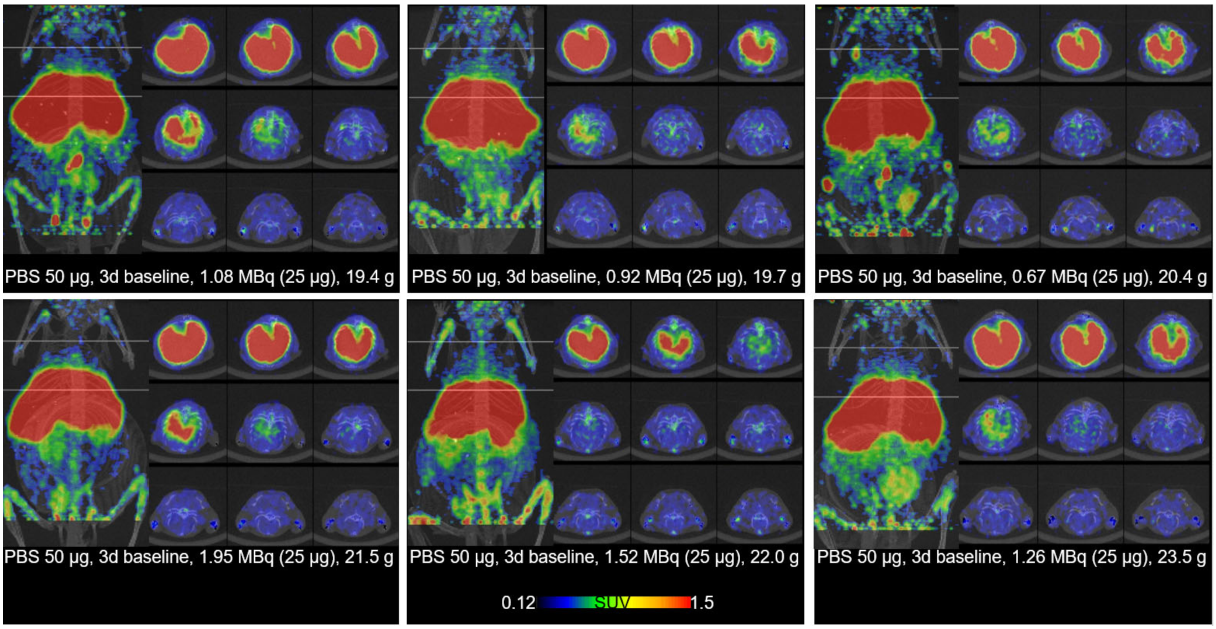

Supporting Figure S9. ^{64}Cu-NODAGA-abatacept PET/CT images 3 d after s.c. PBS/Matrigel inoculation on the right shoulder (as control). ^{64}Cu-NODAGA-abatacept was i.v. injected 48 h before scan start. Dose in MBq at scan start (with corresponding mass in µg) and body weight as indicated. For further details, see Figure 3 in the manuscript. No tracer accumulation was visible in the region of PBS/Matrigel inoculation.

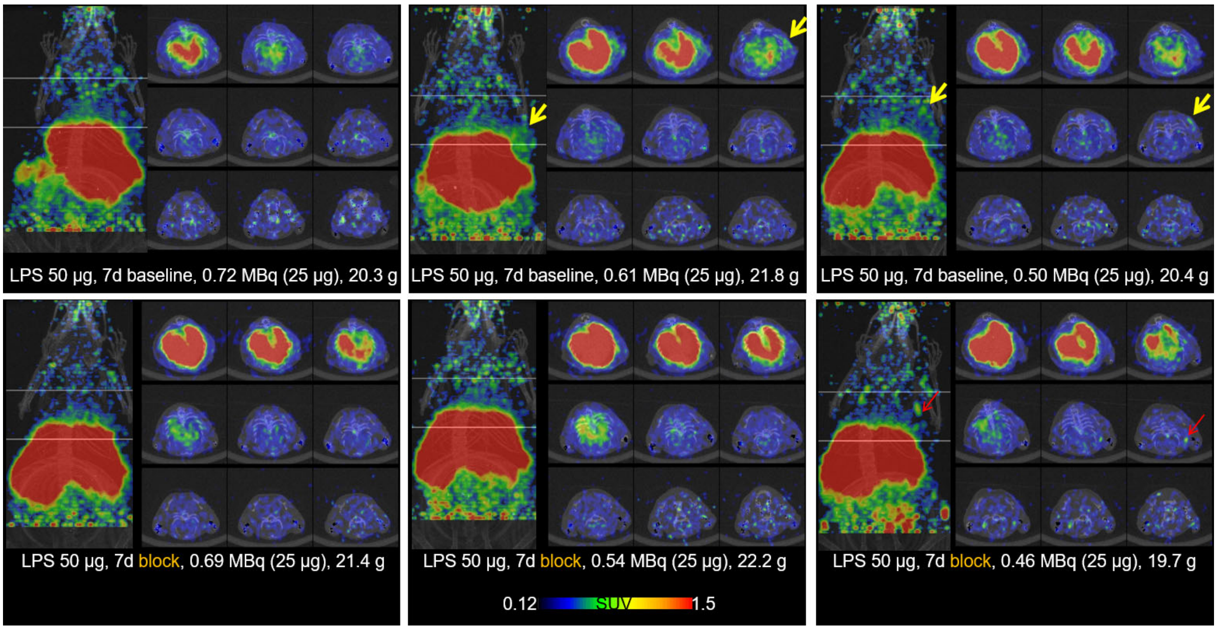

Supporting Figure S10. ^{64}Cu-NODAGA-abatacept PET/CT images 7 d after s.c. LPS/Matrigel inoculation on the right shoulder. ^{64}Cu-NODAGA-abatacept was i.v. injected 48 h before scan start either alone (top row, "baseline") or together with 1 mg unconjugated abatacept for blocking (bottom row, "block"). Dose in MBq at scan start and body weight as indicated. Yellow arrows, tracer accumulation in the region of LPS/Matrigel inoculation. Red arrows, tracer accumulation in the region of lymph nodes. For further details, see Figure 3 in the manuscript.

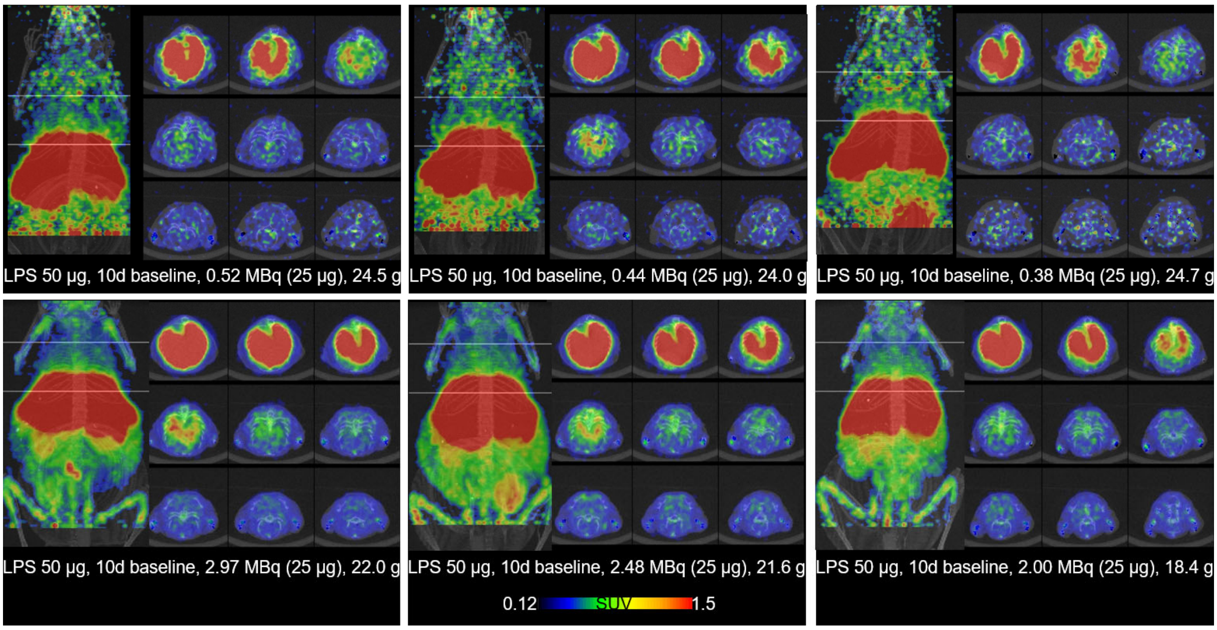

Supporting Figure S11. ^{64}Cu-NODAGA-abatacept PET/CT images 10 d after s.c. LPS/Matrigel inoculation on the right shoulder. ^{64}Cu-NODAGA-abatacept was i.v. injected 48 h before scan start. Dose in MBq at scan start and body weight as indicated. For further details, see Figure 3 in the manuscript.

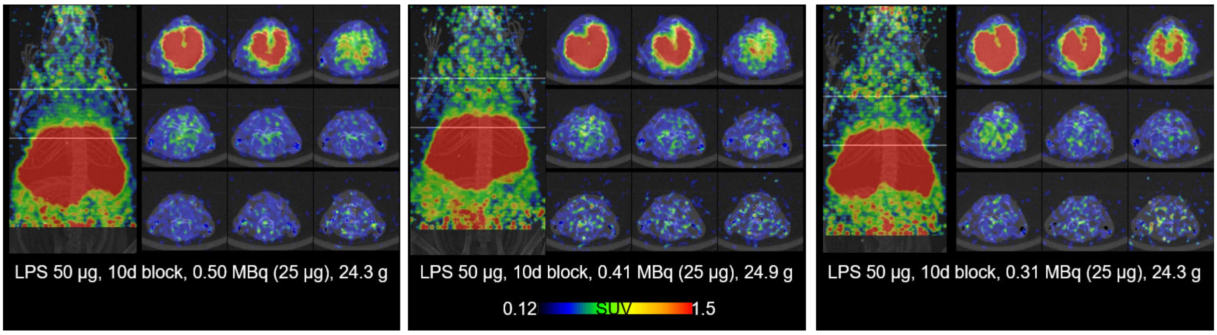

Supporting Figure S12. ^{64}Cu-NODAGA-abatacept PET/CT images 10 d after s.c. LPS/Matrigel inoculation on the right shoulder, under blocking conditions. ^{64}Cu-NODAGA-abatacept was i.v. injected 48 h before scan start together with 1 mg unconjugated abatacept for blocking ("block"). Dose in MBq at scan start and body weight as indicated. Yellow arrows, tracer accumulation in the region of LPS/Matrigel inoculation. For further details, see Figure 3 in the manuscript.

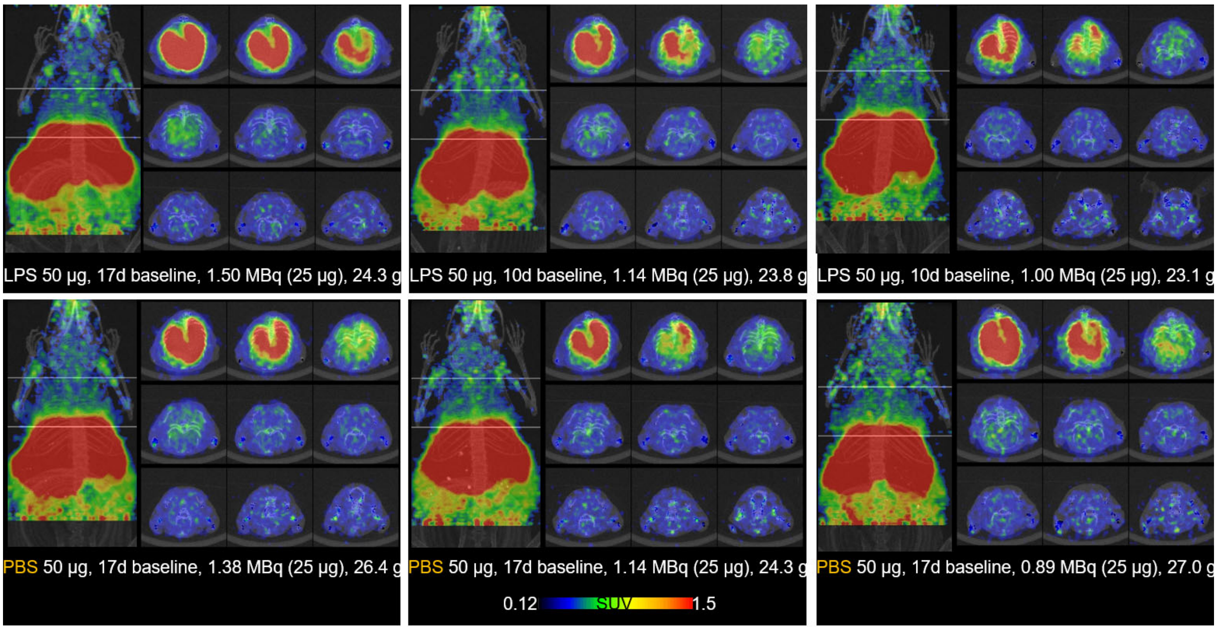

Supporting Figure S13. ^{64}Cu-NODAGA-abatacept PET/CT images 17 d after s.c. LPS/Matrigel inoculation on the right shoulder. ^{64}Cu-NODAGA-abatacept was i.v. injected 48 h before scan start. Dose in MBq at scan start and body weight as indicated. For further details, see Figure 3 in the manuscript.

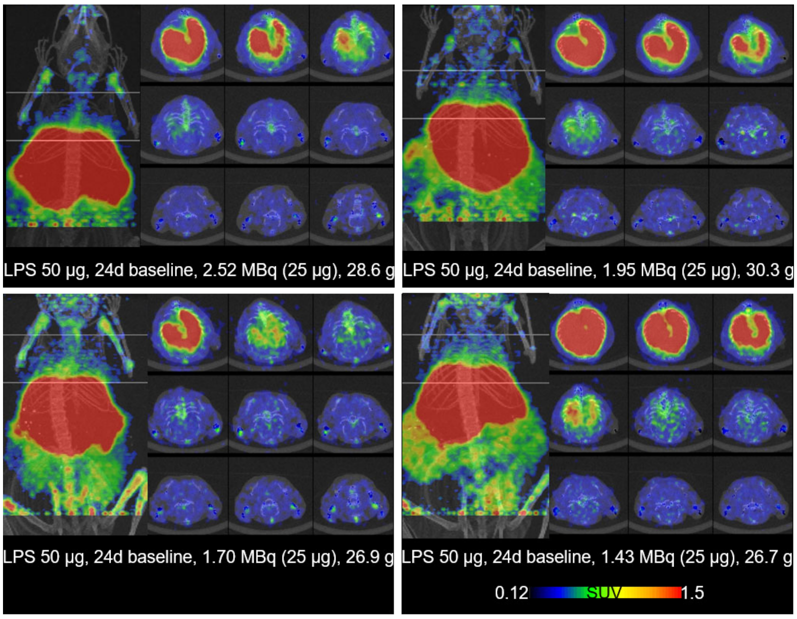

Supporting Figure S14. ^{64}Cu-NODAGA-abatacept PET/CT images 24 d after s.c. LPS/Matrigel inoculation on the right shoulder. ^{64}Cu-NODAGA-abatacept was i.v. injected 48 h before scan start. Dose in MBq at scan start and body weight as indicated. For further details, see Figure 3 in the manuscript.

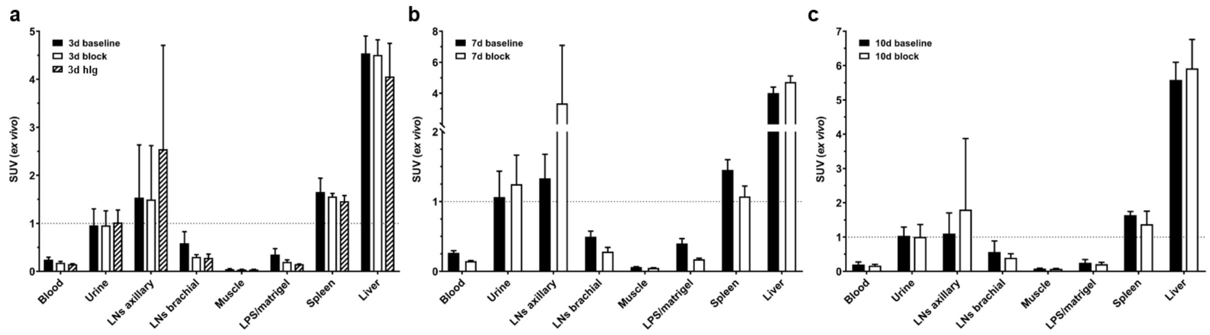

Supporting Figure S15. *Ex vivo* biodistribution data of ^{64}Cu-NODAGA-abatacept 48 h after i.v. injection of radiotracer in mice with s.c. LPS/Matrigel plugs (data as in Figures 4 and 6, with additional tissues). **a**,**b**,**c**) Days after LPS/Matrigel inoculation as indicated. Data shows baseline (tracer only), mice with additional 1 mg unconjugated abatacept ("block") and mice with additional 1 mg hIg ("hIg"), simultaneously injected with the tracer. Horizontal dotted lines indicate SUV = 1 which is equivalent to 4.5 % injected dose per g body weight (calculated with the average body weight of all mice in the study, 22.1 g; note different scales.

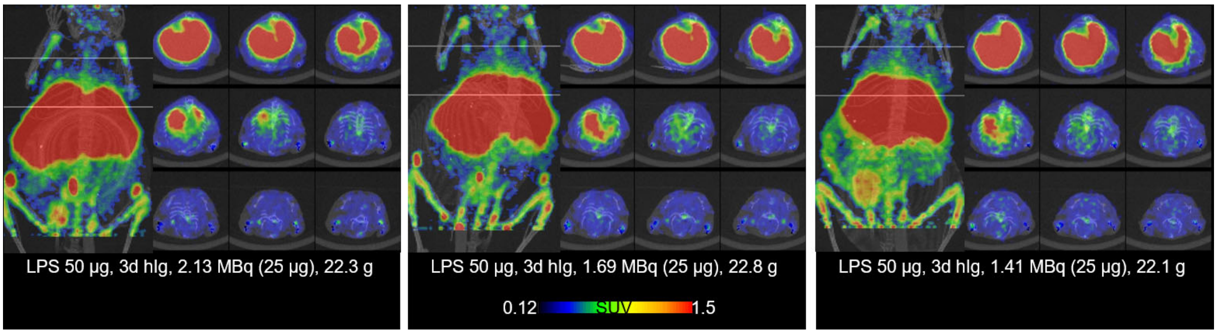

Supporting Figure S16. ^{64}Cu-NODAGA-abatacept PET/CT images in the presence of hIg, 3 d after s.c. LPS/Matrigel inoculation on the right shoulder. ^{64}Cu-NODAGA-abatacept was i.v. injected 48 h before scan start, together with 1 mg hIg. Dose in MBq at scan start and body weight as indicated. For further details, see Figure 3 in the manuscript.

Supporting Tables

Supporting Table S1. Group sizes per condition.

Inoculation	Day	Condition	Analysis [a)]	*n*	Data shown in Figure(s)
**LPS/Matrigel**	3	baseline	PCR, PET	6	2, 3, 4, 5, S7, S15
	3	abatacept (aba)	PET	3	4, S8, S15
	3	hIg	PET	3	6, S16
	3	baseline	FACS	3	1
	3	baseline, aba, hIg	FACS, ELISA	9	5, 6
	6	baseline	PCR	4	2
	6	baseline	FACS	3	1
	7	baseline	PET	3	3, 4, 5, S10, S15
	7	aba	PET	3	3, 4, S10, S15
	10	baseline	PCR, PET	6	2, 4, 5, S11, S15
	10	aba	PET	6	4, S12, S15
	10	baseline	FACS	3	1
	17	baseline	PET	3	4, 5, S13, S15
	17	baseline	FACS	3	1
	24	baseline	PET	4	4, 5, S14, S15
**PBS/Matrigel**	3	baseline	PET	3	3, 4, 5, S9, S15
	3	baseline	FACS, ELISA	3	6
	6	baseline	PCR		2
	17	baseline	PET	3	4, 5, S13
**none**	-	baseline	PET / PCR	4	2, 4, S6, S15
	-	baseline	FACS	3	1

[a)] PET, PET and/or *ex vivo* biodistribution

Supporting Table S2: SPR binding kinetics of abatacept and belatacept to rhCD80, rhCD86 and rmCD80.

**Protein**	**Ligand (*n*)**	K_d **(nM)**	k_a **($M^{-1}s^{-1}$)**	k_d **(s^{-1})**
**hCD80**	Aba (3)	0.668±0.203	1,374,000±268,604	0.000924±0.000333
	NODAGA-Aba (1)	0.647	330,000	0.000212
	Bela (3)	0.259±0.025	1,357,333±140,481	0.000352±0.000052
**hCD86**	Aba (3)	1.178±0.200	1,740,333±290,368	0.002018±0.000311
	Bela (3)	0.408±0.036	2,046,000±163,169	0.000837±0.000108
**mCD80**	Aba (2)	2.64 / 2.31 [a)]	1,020,000 / 1,170,000 [a)]	0.00269 / 0.00271 [a)]
	Bela (2)	11.7 / 12.4 [a)]	207,000 / 242,000 [a)]	0.00240 / 0.00301 [a)]

[a)] for $n = 2$, both experimental values are shown. Aba, abatacept; Bela, belatacept; K_d, dissociation constant; k_a, association rate constant; k_d, dissociation rate constant.
